# Metastatic Basal Cell Carcinoma: A Rare Manifestation of a Common Disease

**DOI:** 10.1155/2017/8929745

**Published:** 2017-11-27

**Authors:** Paola Piva de Freitas, César Galusni Senna, Mayara Tabai, Carlos Takahiro Chone, Albina Altemani

**Affiliations:** ^1^Department of Otolaryngology Head and Neck, Faculty of Medical Sciences, University of Campinas (UNICAMP), Campinas, SP, Brazil; ^2^Department of Pathological Anatomy, Faculty of Medical Sciences, University of Campinas (UNICAMP), Campinas, SP, Brazil

## Abstract

**Introduction:**

Basal cell carcinoma (BCC) is considered the most common malignancy in Caucasians. It constituted about 80% of all nonmelanoma skin tumors and, despite its high prevalence, is an extremely rare occurrence of metastases, with incidence rates varying from 0.0028% to 0.55%.

**Case Report:**

A 58-year-old male patient with BCC on the left nasolabial sulcus for 17 years, reporting 3 previous excisions, evolved with local recurrence. A new procedure was performed, and anatomopathological study confirmed sclerosing BCC. Seven months later, he presented with a mass in the left submandibular region. Combined positron-emission tomography and computed tomography (PET-CT) showed cervical hypercaptation in the left cervical level I and vertebral body of L5. Excision of the cervical lesion was performed with diagnosis of sclerosing BCC compromising the submandibular gland. Biopsy of the lumbar lesion was found to be compatible with bone metastasis.

**Conclusion:**

BCC represents a very common entity, and the presence of metastasis, although infrequent, must be proposed because of the greater morbidity and mortality of this complication. This case shows the importance of early diagnosis and intervention in BCC as a way to avoid unfavorable outcomes.

## 1. Introduction

Basal cell carcinoma (BCC) is considered the most common malignancy in Caucasians. It accounts for about 80% of all nonmelanoma skin tumors, characteristically arising in areas of the body exposed to the sun, its most common location is the head and neck, and it is characterized by slow, locally aggressive growth. Despite its high prevalence, the occurrence of metastases is extremely rare, with incidence rates varying from 0.0028% to 0.55%. The metastases are lymphatic or hematogenic, with regional lymph nodes being the most frequent sites, followed by the lungs and bones. Despite the new treatment options recently developed, the prognosis for metastatic disease remains poor [1, 2].

## 2. Case Report

A 58-year-old male patient presented with a history of basal cell carcinoma on the left nasolabial sulcus for 17 years, having performed three previous excisions with local recurrence. He had a history of intense sun exposure in youth. He did not have primary or acquired immunodeficiency and denied prior radiation therapy or family history of oncology. The patient presented an ulceroinfiltrative lesion of 2.5 cm × 2.0 cm extending through the skin of the maxillary region and the left lateral portion of the nose. There were no palpable cervical masses. Anatomopathological study of the last approach, with deep margin compromised, and computed tomography evidenced infiltration up to periosteum. The patient opted for surgical treatment, so excision of the lesion was performed, with en bloc removal of the maxilla, lateral nasal wall, lateral portion of hard and soft palates on the left, and left neck dissection, levels I to IV. Anatomopathological study confirms the diagnosis of sclerosing basal cell carcinoma, with compromised margin in the region adjacent to the nasal root, perineural carcinomatous invasion, and vascular emboli, and the absence of lymph node metastasis. Posteriorly, he was subjected to adjuvant radiotherapy, presenting clinical remission after treatment.

**Figure 1:**
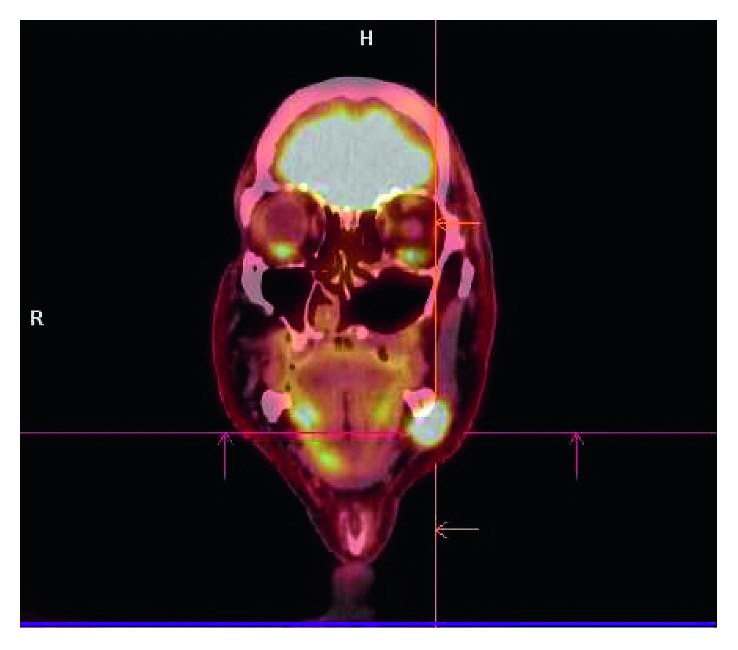
PET-CT shows hypercaptation area of 1.9 cm in the left cervical level I, compatible with metastasis.

**Figure 2:**
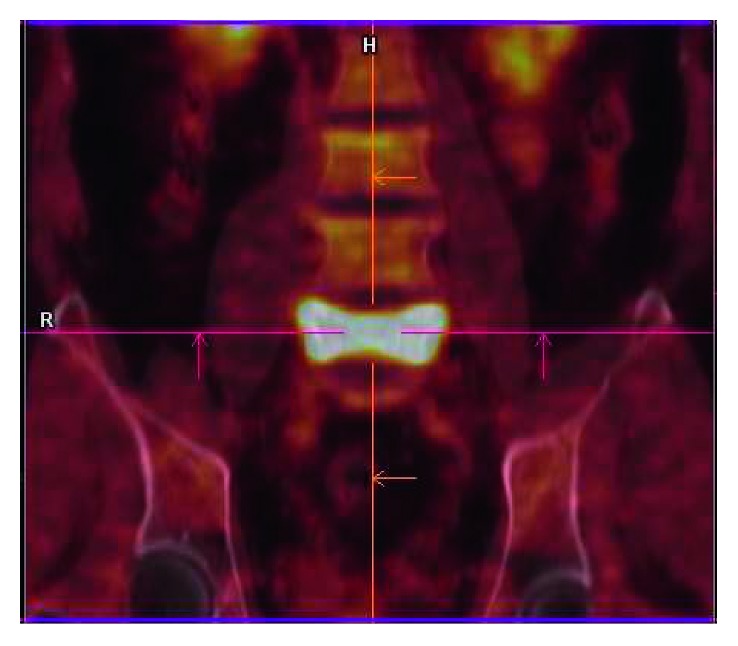
PET-CT shows hypercaptation area on the vertebral body of L5, compatible with bone metastasis.

**Figure 3:**
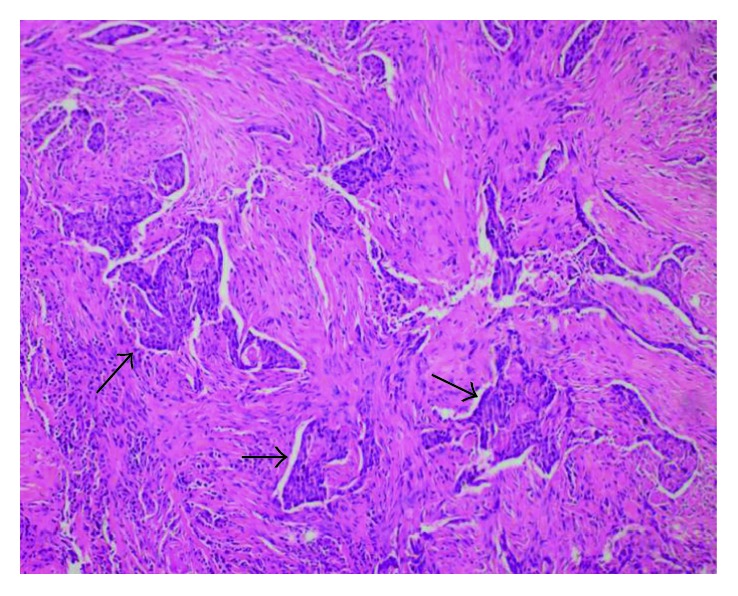
Histological representation of metastasis in the left submandibular gland, showing basaloid cell strings (black arrows), with rounded and hyperchromatic nuclei, compatible with the cellular pattern of the primary site, from between the glandular stromata. The tumor was completely removed for histological analysis.

**Figure 4:**
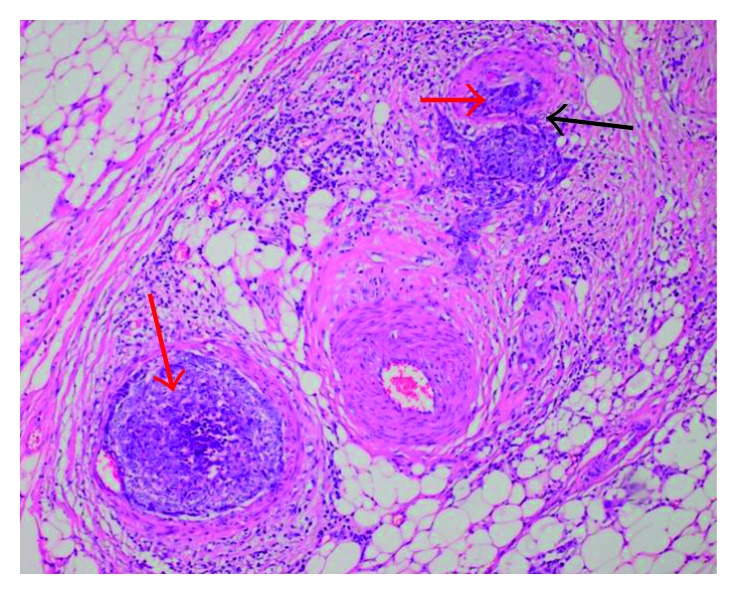
Histological representation of metastasis in the left submandibular gland, showing basaloid cells, compatible with the cellular pattern of the primary site, inside the vessels, provoking tumor thrombi (red arrows). Presence of vascular invasion behavior, indicating probable hematogenous origin of metastases (black arrow).

**Figure 5:**
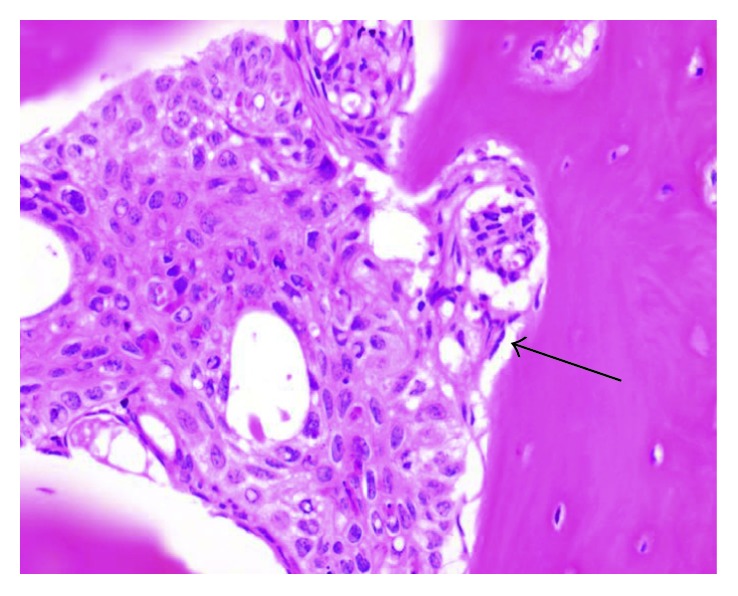
Histological representation of bone metastasis in L5, showing basaloid cell (black arrow), with rounded and hyperchromatic nuclei, compatible with the cellular pattern of the primary site, from between normal bone cells.

At follow-up, 7 months after surgery, he presented with a painful mass with progressive growth in the left submandibular region, adhered to the mandible. PET-CT has showed hypercaptation of 1.9 cm in the left cervical level I and vertebral body of L5, with pathological fracture (Figures 1 and 2). Then, he was subjected to a new procedure, with modified radical neck dissection and marginal mandibulectomy, whose anatomopathological findings indicated sclerosing basal cell carcinoma compromising the submandibular gland and adjacent soft tissues, a very similar pattern to the primary tumor, with perineural and vascular invasion (Figures 3 and 4). Faced with this, metastatic disease was considered. Concomitant to this, a biopsy of the L5 lesion was performed, evidencing a histological appearance very similar to the primary tumor and submandibular lesion, compatible with bone metastasis (Figure 5). Then, he was subjected to lumbar spine arthrodesis.

In postoperative follow-up, a new ulceroinfiltrative lesion was presented in the upper margin of the operative wound, nasal root region, and medial epicanto of the left eye, with induration up to the medial third of the lower eyelid, whose biopsy confirmed local recurrence without surgical proposal. He underwent chemotherapy with paclitaxel and carboplatin, interrupted by toxicity. The patient remains with clinically stable disease 21 months after diagnosis of the first metastasis.

## 3. Discussion

The first case of mastastatic basal cell carcinoma (BCC) was reported by Beadles in 1894 [3]. In 1951, Lattes and Kessler described the most widely accepted criteria for the diagnosis of metastatic basal cell carcinoma (mBCC): (a) the primary lesion and metastasis must have histological confirmation and not be predominantly squamous; (b) the primary tumor must originate from the skin and not from salivary glands or mucous membranes; and (c) direct dissemination of the tumor must be excluded [4].

The current basis for understanding mBCC is derived from two major literature reviews. The first review reported 170 cases from 1894 to 1980, and a subsequent review described 194 cases between 1981 and 2011 [5, 6]. To complement these reviews, we performed a search in the PubMed database, using the terms “basal cell” or “basal cell carcinoma”; “metastatic,” “metastases,” or “metastasis”; and “skin,” covering all publications between January 2012 and March 2017. Cases of BCC were defined using the Lattes and Kessler criteria. Thirty cases were identified, of which 6 were excluded (2 were not in the English or Portuguese language, 3 had no histological evidence of metastasis, and 1 had a basal cell nevus syndrome), resulting in 24 cases. Added to these a case reported in the present study, 25 cases were analyzed in total (Table 1).

In our series, the majority were men with a median age of 65 years, indicating a trend of increased incidence in the male population and onset at a higher age [5–7].

The relationship between the size of the primary tumor and the development of metastases is described: lesions smaller than 3 cm with an incidence of 2%, lesions up to 5 cm with 25%, and tumors greater than 10 cm with 50% [8]. The mean size of the primary tumor in our study was 6.1 cm (*N* = 13, of which two were greater than 10 cm and two were less than 3 cm). In addition to these risk factors, history of previous radiotherapy, local recurrence, location in the central portion of the face or ears, long period of evolution, presence of perineural or perivascular invasion, and possibly immunosuppression are associated with a higher occurrence of metastases [1, 2, 9, 10, 11].

Regarding the location of the primary tumor, the majority were in the head and neck, followed by trunk and extremities, revealing a decrease in the incidence of primary tumors in the head and neck, although this remains the most frequent site [5–7].

The most frequent histological variants were infiltrative, corresponding to 41%, followed by sclerosing, micronodular, and basosquamous/metatypical cell carcinomas in the same proportion, 17% each, and one case of mixed tumor (nodular and infiltrative), corroborating previous studies [2, 12].

The pathways of tumor dissemination described are lymphatic or hematogenic, with the most common site of metastases being lymph nodes, followed by the lungs. Compared with previous studies, there was a decrease in the proportion of lymph node metastases to the detriment of systemic ones [1, 13].

The mean interval between the onset of the tumor and the identification of metastases was approximately 7 years, lower in comparison to the literature, which describes 9 years. [1, 5, 13] Previous studies have indicated a poorer survival, lower in patients with distant metastases when compared to those with only lymph node involvement, estimated at 24 months for the former and 87 months for the latter. Our study does not allow comparisons with these data because of the small proportion of the sample with available prognostic data, that is, only 5 cases [12].

Data available in the literature on the treatment of mBCC are limited, most of which are based on small series and case reports. In general, surgery and radiotherapy are the most common modalities. Platinum-based chemotherapy also showed a favorable response. From 2012 onwards, the inhibitors of the Hedgehog signaling pathway, the first specific therapeutic modality, emerged as an option for patients not candidates for surgery or radiotherapy. They are associated with a high rate of adverse events and low tolerability. The response rates of these new therapeutic modalities for metastatic disease vary between 37% and 15%, thus remaining the restricted prognosis [14–16].

Among the patients analyzed, 92% received some form of treatment, 80% of which underwent surgery, 40% to radiotherapy, and 28% to chemotherapy, of which 60% received combined therapies. Two patients chose not to perform any type of treatment. Only 16% used inhibitors of the Hedgehog signaling pathway, which suggests an underutilization of this new therapeutic modality.

## 4. Conclusion

Basal cell carcinoma represents a very common entity, and the presence of metastasis, although infrequent, must be considered because of the greater morbidity and mortality of this complication. This case shows the importance of diagnosis and early intervention in BCC as the best way to avoid unfavorable outcomes.

## Figures and Tables

**Table 1:** Characteristics of metastatic basal cell carcinoma cases.

Characteristics	Current study (2012–2017)	Wysong et al. (1981–2011)	von Domarus and Stevens (1894–1980)	Comparison of the studies
Total no. of cases	25	194	170	389
Male, no. (%)^a^	20 (80%)	136 (71%)	102 (67%)	258 (66%)
Ratio (male : female)	4 : 1	3 : 1	2 : 1	2 : 1
Age at onset of primary tumor, median (range), y^b^	65 (36–83)	50 (24–88)	45 (14–84)	—

Location of primary tumor				

Head and neck	12 (48%)	122 (64%)	119 (75%)	253 (65%)
Trunk	6 (24%)	41 (21%)	26 (17%)	73 (18.7%)
Extremities	5 (20%)	10 (5%)	11 (7%)	26 (6.6%)
Genitalia	1 (4%)	10 (5%)	2 (1%)	13 (3.3%)
Multiple sites	1 (4%)	9 (5%)	—	10 (2.5%)
Interval from onset of tumor to metastasis, median (range), y^c^	6 (0–18)	9 (0–30)	9 (0–45)	—
Age at first sign of metastasis, median (range), y	69 (40–90)	63 (32–92)	59 (24–89)	—

Site of metastasis^d^				

Lymph nodes	14 (56%)	101 (53%)	63 (39%)	178 (45%)
Lung	9 (36%)	62 (33%)	66 (42%)	137 (35%)
Parotid gland	5 (20%)	—	—	5 (1.2%)
Bone	4 (16%)	38 (20%)	44 (28%)	86 (22%)
Submandibular gland	3 (12%)	—	—	3 (0.7%)
Thyroid	1 (4%)	—	—	1 (0.2%)
Skin/subcutaneous	0	21 (11%)	19 (12%)	40 (10%)
Liver	0	8 (4%)	15 (9%)	23 (6%)
Time to death after diagnosis of metastasis, median (range), mo^e^	24 (6–300)	10 (0.5–108)	8 (1–192)	—

^a^ *N* = 25 for current study, *N* = 191 for study by Wysong et al., and *N* = 153 for study by von Domarus and Stevens. ^b^*N* = 22 for current study, *N* = 157 for study by Wysong et al., and *N* = 130 for study by von Domarus and Stevens. ^c^*N* = 21 for current study, *N* = 152 for study by Wysong et al., and *N* = 137 for study by von Domarus and Stevens. ^d^Forty-seven cases involved more than 1 organ system; *N* = 25 for current study, *N* = 190 for study by Wysong et al., *N* = 159 for study by von Domarus and Stevens. ^e^Cases where survival data are available: *N* = 5 for current study, *N* = 51 for study by Wysong et al., and *N* = 79 for study by von Domarus and Stevens.
